# Plasma Proteins and Cancer

**DOI:** 10.3390/cancers13051062

**Published:** 2021-03-03

**Authors:** Stefan Enroth

**Affiliations:** Department of Immunology, Genetics, and Pathology, Biomedical Center, Science for Life Laboratory (SciLifeLab), Uppsala University, 75185 Uppsala, Sweden; stefan.enroth@igp.uu.se

The human plasma comes into contact with virtually all the cells in the human body and can be easily sampled through phlebotomy. The plasma has been shown to contain thousands of different proteins, and this plasma proteome is further reflective of the individual’s health status. Changes to specific, or combinations of, plasma protein biomarkers in relation to disease can be used in screening and diagnosis or in the monitoring of treatment. In addition to diseases, anthropometrics, genetics and lifestyle as well as technical factors can, however, influence the observed concentrations of the plasma proteins. This Special Issue on plasma proteins and cancer reflects a broad spectrum of disease endpoints, both from a predictive perspective on treatment selection and outcomes and also containing an article on the effects of pre-sampling variance.

Uniform pre-sampling conditions are key for the fair discovery of robust protein plasma biomarkers, and here, Gyllensten and colleagues [1] have examined the differences in the plasma proteome in ovarian cancer samples collected during surgery and before the start of treatment. The samples collected during surgery came from fasting individuals under general anaesthesia, while the other samples were taken at the time of diagnosis. Comparing these groups, their analysis revealed significant differences for about 40% of the 900+ proteins studied, and not accounting for these differences could result in both false positives and false negatives in the discovery of biomarker candidates.

Multi-omics analyses can improve the predictive value of plasma proteins. Two studies in this Special Issue also included genetic information, either by SNP genotyping or cell-free DNA detection through next-generation sequencing. The work by Drobin and colleagues [2] used genetic markers and plasma proteins to build multivariate models predicting radiosensitivity in patients with breast or head-and-neck cancer. Interestingly, a single model consisting of eight proteins and one genetic marker performed well for both cancers, indicating that different cancers result in the same downstream effects on the plasma proteome, which is indicative of underlying radiosensitivity. Additionally using multi-omics, Steendam and colleagues [3] investigated biomarkers for nonresponsiveness to treatment against mutated EGFR non-small-cell lung cancers. Finding predictive markers for this condition early in the disease’s progression is important, as nonresponsive patients have poor prognosis and need to be closely monitored and possibly change treatment regime.

Using multivariate models based on plasma proteins alone, Purohit and colleagues [4] examined survival and therapeutic responses among patients with cervical cancer. The functional analysis of the proteins in their modelling revealed associations with cellular senescence and that this, in turn, could be used to guide whether brachytherapy would be effective or not for the individual patient.

This Special Issue also includes an in-depth review by Conteduca and colleagues [5] on the role of the androgen receptor in prostate cancer. There, it is clear from the existing evidence that this role changes with the progression of the disease, and different aspects, such as underlying genetic mutations, different splice variants and copy number variation of the androgen receptor, are detectable in the plasma. The authors conclude that the “integration of different biomarker strategies, including genomics, with plasma AR status in prostate cancer, could substantially improve prognostication and stratification of these patients”.

This also holds true in a larger perspective, for other biomarkers and for the other cancer types studied in this Special Issue. Combinations of plasma protein biomarkers alone or in conjunction with other types of data such as genetic variants or cell-free DNA as described here show promising results in relation to treatment guidance and prognosis. This is enabled by the use of high-throughput technologies in combination with sophisticated analysis of often-multi-omics data in relation to specified outcomes in large cohorts. Blood sampling is minimally invasive for the patient and can be used with a large battery of analysis methods but still requires trained medical personnel and downstream infrastructure for the separation of, for example, plasma and biobanking. An existing alternative to conventional phlebotomy is the collection of capillary blood as dried blood spots, which can be performed by the individuals themselves, even at home. Although the field of proteomics from dried blood spots is largely unexplored and several outstanding questions remain, combinations of plasma biomarkers for treatment monitoring as presented in this Special Issue and the concept of at-home blood sampling truly open up new research directions for the future of the cost-efficient use of cancer biomarkers.

## References

[1] Gyllensten U., Bosdotter Enroth S., Stålberg K., Sundfeldt K., Enroth S. (2020). Preoperative Fasting and General Anaesthesia Alter the Plasma Proteome. Cancers.

[2] Drobin K., Marczyk M., Halle M., Danielsson D., Papiez A., Sangsuwan T., Bendes A., Hong M.-G., Qundos U., Harms-Ringdahl M. (2020). Molecular Profiling for Predictors of Radiosensitivity in Patients with Breast or Head-and-Neck Cancer. Cancers.

[3] Steendam C.M.J., Veerman G.D.M., Pruis M.A., Atmodimedjo P., Paats M.S., van der Leest C., von der Thüsen J.H., Yick D.C.Y., Oomen-de Hoop E., Koolen S.L.W. (2020). Plasma Predictive Features in Treating EGFR-Mutated Non-Small Cell Lung Cancer. Cancers.

[4] Purohit S., Zhi W., Ferris D.G., Alverez M., Tran L.K.H., Tran P.M.H., Dun B., Hopkins D., Santos B.D., Ghamande S. (2020). Senescence-Associated Secretory Phenotype Determines Survival and Therapeutic Response in Cervical Cancer. Cancers.

[5] Conteduca V., Gurioli G., Brighi N., Lolli C., Schepisi G., Casadei C., Burgio S.L., Gargiulo S., Ravaglia G., Rossi L. (2019). Plasma Androgen Receptor in Prostate Cancer. Cancers.

